# Genetic Parameters for Determining Useful Parents in Mungbean (V*igna radiata* (L.) Wilczek) Breeding for Early Maturity, Small Seed Size, and High Seed Yield

**DOI:** 10.1155/2021/3910073

**Published:** 2021-12-31

**Authors:** Trustinah, Rudi Iswanto, Ratri T. Hapsari, Novita Nugrahaeni, Rudy Soehendi, Titik Sundari, Suhartina, Febria C. Indriani, Made J. Mejaya

**Affiliations:** Indonesian Legumes and Tuber Crops Research Institute (ILETRI), Jl. Raya Kendalpayak Km 8, P.O. Box 66, Malang, East Java, Indonesia

## Abstract

Early maturity, small seed size, and high seed yield are important characters of mungbean in Indonesia. The objective of the study was to determine the useful parents in mungbean crosses for early maturity, small seed size, and high seed yield varieties by estimating the genetic parameters and their inheritance. The study was conducted at the ILETRI, Malang, East Java, Indonesia, during the dry season of 2014. 20 F1 and 5 parents were evaluated using a randomized block design, repeated three times. Results of the study showed that all observed traits showed the importance of both additive and dominance gene effects. The relative value of general combining ability (GCA) was greater than specific combining ability (SCA) for number of pod clusters per plant, number of branches per plant, plant height, days to maturity, and 100-seed weight which indicated the importance of additive gene effect. The dominance gene effect occurred on number of pods and seed yield per plant. Among five parents, G3 was the best combiner for all the observed characters except pod length; therefore, G3 could be exploited for late maturity, small seed size, high number of branches and pod cluster, and high seed yield. G5 has a high GCA for 100-seed weight. G1 and G2 have good GCA for early maturity. G3 and G5 genotypes are useful as parents in mungbean breeding for small and large seed size varieties, respectively. The best combination for seed yield was G2 × G3 and G3 × G1 crosses and could be proceeded with selection for early maturity, small seed size, and high seed yield varieties.

## 1. Introduction

Mungbean (*Vigna radiata* (L.) Wilczek) is the third most important legumes in Indonesia after soybean and peanut. Mungbean seeds as other legume crops contain a good concentrations of minerals, vitamins, and antioxidants to overcome the malnutrition problems [1] and effect of heavy metal contamination [2].

The harvested area of mungbean in Indonesia in 2018 was 190,000 ha and the production reached 207,169 tonnes with an average productivity of 1,079 t ha^−1^ ranging from 0,843 to 1,453 t ha^−1^. In Indonesia, mostly mungbeans are grown in paddy fields and dry land. In paddy fields, mungbean is usually cultivated after lowland rice in the dry season or grown as the third crop on the cropping pattern rice-rice-mungbean or rice-soybean/maize-mungbean. Mungbeans on dry land are usually planted after other upland crops [3].

The mungbean characteristics needed for the component of cropping pattern are early maturity, drought resistance, and pest and disease resistance. In areas with limited labor, it is very important to have mungbean varieties with synchronous pod maturity characteristics. Small seed varieties are commonly used for sprouts industry to obtain more sprouts per kg of seeds. Large seed size varieties are used for food and beverage industries made from mungbean. More than 20 improved varieties of mungbeans have been released in Indonesia with various characteristics (seed color is dull green or shiny green, and seed size is small to large), yields range from 0.90 to 1.98 t ha^−1^, 100-seeds weight 2.5 g (small seed size) to 7.8 g (large seed size), and maturity of 51 days after planting (early maturity) to more than 70 days after planting (late maturity) [4]. Early maturity and high quality seed characters as well as improved agronomic practices are important to enhance mungbean productivity [5].

Improving the existing varieties is a common activity in breeding program by creating genetic diversity [6]. In the current study, Sampeong mungbean variety was improved by making crosses with two new varieties (Vima 1 and Vima 2) and the promising lines. Determining useful parents for crosses is a critical step in breeding programs. By predicting the genetic parameters of certain characters of the genotypes, therefore, the useful parents could be determined for crosses. The next step is a selection method to develop the improved varieties in breeding. Diallel analysis could be used to study the genetic basis of a character. This analysis method can predict the additive, dominant, and genetic variability and heritability values of each character in the observed population. Several methods have been developed for diallel analysis. Estimation of genetic parameters and inheritance certain characters using diallel analysis has been carried out on several legume crops such as mungbeans [7–11], soybeans [12], snap beans [13], and peanuts [14].

The objective of the study was to determine the useful parents in mungbean crosses for early maturity, small seed size, and high seed yield varieties by estimating the genetic parameters and their inheritance.

## 2. Materials and Methods

### 2.1. Study Sites

This study was conducted during dry season of 2014 at the Indonesian Legumes and Tuber Crops Research Institute (ILETRI), Malang, East Java, Indonesia (8° 2′ 56.4″ S and 112° 37′ 30″ E; 445 meters above sea level).

### 2.2. Plant Materials

The experimental material consisted of five mungbean genotypes (MMC 679-3C-GT-1 (G1), MMC 672-3C-GT-1 (G2), Sampeong (G3), Vima 2 (G4), and MLG 1065 (G5) which were phenotypically different.  Table 1 shows the characteristics of parents used for diallel crosses for developing small-seeded mungbean improved varieties.

The 5 × 5 complete diallel trial was evaluated using a randomized block design, repeated three times. The treatments were 20 F1 series and 5 parents. Each plot consisted of 30 plants, spacing of 40 cm between rows, 10 cm in the row, and one seed per hole resulting in 250,000 plants per hectare. The crops were fertilized with 50 kg Urea, 100 kg SP36, and 50 kg KCl per hectare and were applied at sowing time. All the recommended agronomic practices were done.

### 2.3. Data Collection

Observations were made on all individual plants for number of pod clusters per plant, number of branches, plant height (cm), number of pods per plant, pod length (cm), days to maturity, 100-seed weight (g), and seed yield per plant (g).

### 2.4. Data Analysis

Analyses of variance were carried out for each trait, according to the model:

(1) $$\begin{array}{c} Y_{ij}=\mu +g_{i}+b_{j}+\varepsilon_{ij}. \end{array}$$

Here, *Y*_*ij*_ is the value measured in the plot, analyzed for the *i*-th genotype, in the *j*-th block; *μ* is the overall mean; *g*_*i*_ is the effect of the *i*-th genotype, considered as fixed (*i* = 1, 2,…, 25); bj is the effect of the *j*-th block, considered as fixed (*j* = 1, 2,…, 5); *e*_*ij*_ is the effect of the random error associated with the *ij* observation with ∼NID (0, *σ*^2^).

Diallel analyses were carried out according to model 1 of Griffing [4]:

(2) $$\begin{array}{c} Y_{ij}=\mu +g_{i}+g_{j}+s_{ij}+r_{ij}+\varepsilon_{ij}. \end{array}$$

Here, *Y*_*ij*_ is the mean value of the hybrid combination (*i* ≠ *j*) or of the parent (*i* = *j*); *μ* is the general mean; *g*_*i*_ and *g*_*j*_ are the effects of the general combining ability of the *i*-th genotype or of the *j*-th parent (*i*, *j* = 1, 2, and 3); sij is the effect of the specific combining ability for crosses between parents *i* and *j*; *r*_*ij*_ is the reciprocal effect that measures the difference provided by parent *i* or *j* when used as male or female in the cross *ij*; and *e*_*ij*_ is the effect of the mean error associated with the *ij* observation with ∼ NID (0, *σ*^2^). In this model, the gi effect was defined as *i* ranging from 1 to number of genotypes (five), since the diallel analysis was performed using the experimental unit mean (thirty plants per experimental unit). It was considered *s*_*ij*_ = *s*_*ji*_, *r*_*ij*_ = − *r*_*ji*_, and *r*_*ii*_ = 0. From this model, estimates of general combining ability (GCA) and of specific combining ability (SCA), and reciprocal effects, were obtained. Data were analyzed using the PBTools version 1.4 [15].

## 3. Results and Discussion

### 3.1. Analysis of Variance

The analysis of variance for eight mungbean characters is shown in Table 2. There was a highly significant difference in variance for all characters (number of pod clusters per plant, number of branches, plant height, number of pods, plant age, weight of 100 seeds, and yield of seeds per plant) indicating the diversity of the test material, both parents and their respective crosses. Therefore, the estimation of combining ability using diallel analysis can be performed.

The analysis of general combining ability (GCA), specific combining ability (SCA), and reciprocal for eight mungbean characters is presented in Table 2. The mean squares of GCA and SCA were significant for number of pod clusters, number of branches, plant height, number of pods per plant, days to maturity, and seed yield per plant. While pod length was only significant for SCA, the mean square of the reciprocal was not significant for all of the observed traits indicating no influence of the female parent on the inheritance of these trait (Table 2). The significant mean squares of GCA and SCA indicated the importance of the effects of additive and dominant genes, while pod length was only significant for SCA, indicating the importance of the influence of nonadditive genes. The significant effects of general combining ability and specific combining ability on mungbean for plant height, number of pods, days to maturity, seed yield, and weight of 100 seeds were also reported by [5, 6, 16, 17].

### 3.2. Type of Gene Action

The effect of additive and dominant genes for each character is indicated by their magnitude estimates of variance components GCA and SCA, the narrow and broad sense heritability, and the dominance ratio. Some authors have used the variance component ratio of combining ability (predictability ratio) (2*σ*^2^ GCA/2*σ*^2^ GCA+*σ*^2^SCA) to determine the type of gene action affected traits expression. The predictability ratio greater than 0.5 indicated additive gene action, less than 0.5 indicated nonadditive gene action, and 0.5 indicated predominance of additive and nonadditive gene action for a character [18–20].

In the present study, the predictability ratio ranged from 0.05 to 0.97. Number of pod clusters per plant, number of branches per plant, plant height, days to maturity, and weight of 100 seeds had a predictability ratio greater than 0.5 indicating the action of additive genes. On the other hand, the number of pods, pod length, and seed yield had a predictability ratio of less than 0.5 indicating the predominance of nonadditive gene action. The weight of 100 seeds had the highest predictability ratio of 0.97 and the lowest was the seed yield with a predictability ratio of 0.25 (Table 3). The converse results for the predictability ratio of 100-seed weight and grain yield were reported by Nath et al. [20].

The weight of 100 seeds had high estimates of genetic variance component of GCA indicated by the highest ratio of GCA and SCA variance among the eight observed traits, high values of narrow sense (0.92) and broad sense (0.94) heritabilities, and low dominance ratio (0.17) (Table 3). These results agree with those reported by Jambormias et al. [21] and Kajonphol et al. [22]. The high influence of additive genes will make it easier for character improvement through selection of segregated population.

The seed yields showed that SCA variance was greater than GCA variance and predictability ratio (0.40), low narrow sense heritability (0.37), and high dominance ratio (1.72) indicated the dominance of nonadditive genes. The same results were seen for the pod length (Table 3). SCA variance greater than the GCA variance for seed yield/plant and number of pods/plant were also reported by Zubair et al. [16], Patil et al. [5], Selvam [6], and Shrinkhala et al. [23]. A dominance ratio greater than 1 (one) indicated a greater proportion of dominant genes than recessive genes [24].

The greater SCA variance suggests that selection for these traits cannot be made in the early generations [6, 16, 25]. The present results were in congruent with the reports of Selvam [6] and Khaimichho [26]. Different results were reported by Sopan et al. [9] and Shrinkhala et al. [23]. These differences can be due to the genetic background of the varieties used in various studies and the environment in which the experiments were conducted, because polygenic characters like seed yield are more controlled by environmental fluctuations. The number of parents were used in the diallel analysis also determines the ratio of the mean square GCA and SCA [27].

### 3.3. Mean Performance of Agronomic Characters

The mean performance of eight agronomic characters in 5 × 5 complete diallel of mungbean is presented in Table 4. Among the parents, G3 gave the highest yield for seed yield/plant, number of clusters, number of branches, plant height, number of pods, late maturity, and smallest seed size. G5 had the longest pod and weight of 100 seeds. G1, G2, and G4 had lower plant height than G3 and G5. The highest seed yield/plant was obtained from crossing G2 × G3 and G3 × G1, with seed yield/plant of 8.0 g and 7.18 g, respectively, and 100-seed weight 3.97 g and 5.17 g, respectively. The seed yields/plant of G2 × G3 and G3 × G1 were higher than their respective parents (G2, G3, and G4).

The maturity of the crosses ranged from 56.4 to 61.4 days after planting (Table 4). The single cross G3 × G4 showed the earliest maturity (56.4 days) than its two parents, while G4 × G5 had a late maturity (66 days) which was longer than its two parents. The plant height ranged from 41.67 to 81.80 cm. G3 showed the highest plant height (81.8 cm) and the smallest seed size (3.31 g/100 seeds). Crosses with G3 produced plants with earlier maturity days and larger seed size than G3 parents. Small seed sizes (<4 g/100 seeds) were obtained from crossing G2 × G3 and its reciprocal. The F1 seed size was larger than parent G3 and smaller than parent G2 (Table 4).

### 3.4. General Combining Ability

Analysis of variance showed that the mean squares of general combining ability (GCA) and specific combining ability (SCA) were significant for all the observed traits. Thus, the effects of GCA and SCA for individual lines can be calculated. Among the parents, G3 had good general combining ability for all the observed traits except for pod length, and this indicated that these parents could be exploited for late maturity, small seed size, high yield, high plant height, and high number of branches and pod clusters. G5 has a high general combining ability for the weight of 100 seeds (Table 5).

Both G3 and G5 genotypes are prospective to be used as parents in the mungbean improvement program of small and large seed varieties. G1 and G2 have good general combining ability for early maturity. The genotype with the highest mean value for a character tends to have the maximum GCA for that trait. For example, parent Sampeong (G3) exhibited positive and significant GCA effect and had high mean value for seed yield per plant, number of branches per plant, number of clusters per plant, and number of pods per plant. These findings agreed with the reports of Zubair et al. [16] and Latha et al. [17].

### 3.5. Specific Combining Ability

The results of this study indicated that the parent with the highest GCA effect does not always produce the highest specific cross combination. Of the 20 series of crosses, the best combination for seed yield was the G2 × G3 and G3 × G1 crosses, which were a combination of high GCA *x* low GCA lines for high yields and small seed sizes. The combination had the special combining ability desired for high yield and small seed size (Table 6). The same thing were also reported by El- Badawy [28] and Iriany et al. [29] where cross pairs showing high SCA are a combination of parents with high GCA and low GCA for several important characters. The dominant genetic variances that affect the SCA of a cross pair are thought to be owned by the parents. The dominant alleles combine and interact positively so as to produce a genotype with high combining ability in the F1 results of these crosses.

Based on the general combining ability and the specific combining ability in these crosses, the importance of the effects of additive and nonadditive genes in the inheritance of most of the polygenic traits in mungbeans could be seen. When the GCA variances was higher than SCA variances, early generation selection of genotypes becomes more efficient, while in the presence of nonadditive component, selection should be undertaken in later generations when these impacts are fixed in the homozygous lines [19, 30]. Maximum yield can be achieved with a system that can exploit the effects of additive and nonadditive genes simultaneously. According to Patil et al. [5] in such a situation, mating selected plants in the initial segregation generation can assist in developing potentials that have the desired combination of genes for high yielding.

## 4. Conclusions

All the observed traits were influenced by both additive and nonadditive gene effects. The highest effect of additive gene action was on the weight of 100 seeds, while the seed yield was strongly influenced by the dominance of nonadditive gene action. Among the parents, G3 was the best combiner for all the observed characters except pod length, and this indicated that these parents can be exploited for late maturity, small seed size, high yield, high plant performance, and high number of branches and clusters. G5 has a high general combining ability for weight of 100 seeds. Both of these G3 and G5 lines are prospective to be used as parents in the mungbean improvement of small and large seed mungbean varieties. G1 and G2 have good general combining ability for early maturity. The best combination for seed yield was G2 × G3 and G3 × G1 crosses and could be proceeded with selection for early maturity, small seed size, and high seed yield varieties.

## Figures and Tables

**Table 1:** Characteristics of parents used for diallel crosses of mungbean varieties.

Parent code	Parents	Characters^a)^
G1	MMC 679-3C-GT-1	Early maturity, short plants, medium seed size, green hypocotyl, and dull green seed color
G2	MMC 672-3C-GT-1	Early maturity, short plant performance, medium seeds size, green hypocotyl, and shiny green seed color
G3	Sampeong	Late maturity, high plant performance, small seed size, shiny green seeds color, and purple hypocotyl
G4	Vima 2	Early maturity, resistant to soilborne diseases, medium seed size, green hypocotyl, and shiny green seed color
G5	MLG 1065	Medium maturity, large seed size, and dull green seed color

^a)^Maturity: early maturity = < 60 days after planting; late maturity = > 70 days after planting. Seed size: small seed size = < 3 g of 100-seeds weight, large seed size = > 6 g of 100-seeds weight.

**Table 2:** Analysis of variance of mungbean agronomic characters in 5 × 5 complete diallel.

Source of variation	df.	Mean squares							
		∑ cluster	∑ branches	Plant height	∑ pods/plant	Pod length	Days to maturity	Seed yield per plant	100-seed weight
Replications	2	4.95 ns	0.25 ns	177.50 ^*∗∗*^	69.41 ^*∗∗*^	0.61 ^*∗∗*^	3.36 ns	15.66 ^*∗∗*^	0.48 ns
Genotypes	24	3.76 ^*∗∗*^	0.43 ^*∗∗*^	321.04 ^*∗∗*^	59.33 ^*∗∗*^	0.92 ^*∗∗*^	18.70 ^*∗∗*^	8.62 ^*∗∗*^	4.12 ^*∗∗*^
Error	48	0.28	0.09	10.45	3.69	0.18	4.56	0.73	0.32

Symbols ^*∗*^ and ^*∗∗*^ are significant at 5 and 1% level of probability; ns: nonsignificant.

**Table 3:** Means squares for combining ability and genetic components of variance in 5 × 5 parent complete diallel of mungbean.

Source of variation	df.	Mean squares							
		∑ cluster	∑ branches	Plant height	∑ pods/plant	Pod length	Days to maturity	Seed yield per plant	100-seed weight
GCA	4	6.37 ^*∗∗*^	0.60 ^*∗∗*^	533.42 ^*∗∗*^	82.99 ^*∗∗*^	0.58 ns	22.42 ^*∗∗*^	8.73 ^*∗*^	7.70 ^*∗∗*^
SCA	10	0.44 ^*∗∗*^	0.08 ^*∗*^	36.92 ^*∗∗*^	13.63 ^*∗∗*^	0.47 ^*∗∗*^	3.75 ^*∗*^	3.09 ^*∗∗*^	0.14 ns
Reciprocal	10	0.03 ns	0.03 ns	6.55 ns	0.64 ns	0.04 ns	2.24 ns	0.30 ns	0.07 ns
Error	48	0.09	0.03	3.49	1.23	0.04	1.51	0.24	0.11
Estimates of variance components									
*σ* ^2^ gca		0.59	0.05	49.81	6.99	0.01	1.88	0.58	0.76
*σ* ^2^ sca		0.20	0.03	19.90	7.38	0.25	1.33	1.70	0.02
*σ* ^2^ reciprocal		0.00	0.00	1.53	0.00	0.00	0.37	0.03	0.00
*σ* ^2^ gca/*σ*^2^ sca		2.94	1.74	2.50	0.95	0.05	1.41	0.34	34.2
(2*σ*^2^ gca)/(2*σ*^2^ gca +* σ*^2^ sca)		0.85	0.78	0.83	0.65	0.10	0.73	0.40	0.98
Estimates of genetic variance components									
VA		1.19	0.10	99.62	13.99	0.05	3.76	1.15	1.51
VD		0.20	0.03	19.90	7.38	1.02	1.33	1.70	0.02
H2-narrow sense		0.80	0.64	0.81	0.62	0.05	0.57	0.37	0.92
H2-broad sense		0.94	0.82	0.97	0.95	0.96	0.77	0.92	0.94
Dominance ratio		0.58	0.76	0.63	1.03	6.14	0.84	1.72	0.17

Symbols ^*∗*^ and ^*∗∗*^ are significant at 5 and 1% level of probability; ns: nonsignificant.

**Table 4:** Mean performance for eight agronomic characters in 5 × 5 parents complete diallel of mungbean.

No.	Genotypes	∑ cluster	∑ branches	Plant height (cm)	∑ pods/plant	Pod length (cm)	Days to maturity (DAP)	Seed yield per plant (g)	100-seed weight (g)
*Parents* ^a)^									
1	G1	3.37	0.20	44.80	10.87	8.97	57.37	5.46	5.95
2	G2	4.37	0.13	44.53	12.97	8.93	56.40	4.71	5.69
3	G3	7.93	1.47	81.80	21.13	8.20	63.50	7.37	3.31
4	G4	3.37	0.73	43.77	10.87	9.63	57.13	6.79	6.74
5	G5	3.40	0.33	59.10	13.43	10.17	57.60	6.56	7.76

*Crosses*									
6	G1 × G2	3.47	0.23	47.27	11.53	9.23	59.10	5.26	6.45
7	G1 × G3	4.73	0.77	68.53	16.40	8.57	61.37	6.74	4.54
8	G1 × G4	3.57	0.17	51.17	11.23	9.27	59.93	5.65	5.84
9	G1 × G5	3.60	0.40	47.17	8.10	8.37	60.00	4.19	7.35
10	G2 × G3	5.60	0.83	61.00	21.03	8.83	60.30	8.00	3.97
11	G2 × G4	3.70	0.40	46.03	12.47	9.37	59.60	5.41	6.25
12	G2 × G5	3.60	0.33	45.50	8.10	8.57	58.33	3.11	6.20
13	G3 × G4	3.47	1.37	64.57	11.70	9.20	56.40	5.24	4.74
14	G3 × G5	5.50	0.40	53.17	16.80	7.93	61.23	7.02	5.30
15	G4 × G5	4.17	0.27	43.90	8.37	8.10	66.33	2.86	7.05
16	G2 × G1	4.70	0.13	46.03	16.30	9.10	58.27	6.69	6.24
17	G3 × G1	5.70	0.33	61.30	18.73	8.40	61.40	7.18	5.17
18	G3 × G2	3.27	0.80	63.33	11.53	8.47	57.23	6.29	3.98
19	G4 × G1	3.40	0.23	47.60	11.77	9.40	58.43	5.70	6.08
20	G4 × G2	5.27	0.27	45.43	17.53	9.37	59.87	5.03	6.33
21	G4 × G3	3.50	1.03	63.53	6.67	8.97	60.00	2.79	4.69
22	G5 × G1	3.27	0.20	41.33	5.70	8.20	57.00	2.69	7.25
23	G5 × G2	3.50	0.13	41.67	7.60	8.27	57.00	3.33	7.05
24	G5 × G3	3.67	0.13	56.10	8.90	8.37	64.00	3.68	5.30
25	G5 × G4	3.47	0.57	43.43	6.80	8.53	60.00	3.74	7.30

^a)^ G1 = MMC 679-3C-GT-1, G2 = MMC 672-3C-GT-1, G3 = Sampeong, G4 = Vima 2, G5 = MLG 1065.

**Table 5:** General combining ability effect in 5 × 5 complete diallel of mungbean.

Parents	∑ cluster	∑ branches	Plant height	∑ pods/plant	Pod length	Days to maturity	Seed yield per plant	100-seed weight
G1	−0.46 ^*∗∗*^	−0.19 ^*∗*^	−2.48 ^*∗∗*^	−0.84	0.03	−1.11 ^*∗∗*^	−0.01	0.21
G2	−0.03	−0.13	−3.95 ^*∗∗*^	0.62	0.09	−1.18 ^*∗∗*^	−0.12	−0.06
G3	1.38 ^*∗∗*^	0.39 ^*∗∗*^	13.03 ^*∗∗*^	4.37 ^*∗∗*^	−0.30 ^*∗∗*^	2.46 ^*∗∗*^	1.11 ^*∗∗*^	−1.42 ^*∗∗*^
G4	−0.30 ^*∗*^	0.10	−3.16 ^*∗∗*^	−0.61	0.33 ^*∗*^	−0.46	0.44 ^*∗*^	0.32 ^*∗*^
G5	−0.59 ^*∗∗*^	−0.16 ^*∗*^	−3.44 ^*∗∗*^	−3.55 ^*∗∗*^	−0.15	0.27	−0.43	0.95 ^*∗∗*^
SE (Gij)	0.09	0.05	0.53	0.31	0.06	0.35	0.14	0.09
SE (Gi-Gj)	0.14	0.08	0.83	0.50	0.09	0.55	0.22	0.15
CD at 5%	0.28	0.15	1.68	1.00	0.18	1.11	0.44	0.29

Symbols ^*∗*^ and ^*∗∗*^ are significant at 5 and 1% level of probability.

**Table 6:** Specific combining ability and reciprocal effect in 5 × 5 complete diallel of mungbean.

Crosses	∑ cluster	∑ branches	Plant height	∑ pods/plant	Pod length	Days to maturity	Seed yield per plant	100-seed weight
*Specific combining ability effect*								
G1 × G2	−0.19	0.31	0.60	−0.43	0.23	0.54	0.00	0.34
G1 × G3	−0.34 ^*∗∗*^	−0.12 ^*∗∗*^	1.89 ^*∗∗*^	0.55 ^*∗∗*^	−0.06	−1.06 ^*∗*^	0.23 ^*∗*^	0.20 ^*∗∗*^
G1 × G4	0.04	−0.19	2.55	0.57	0.16	0.62	0.16	−0.44
G1 × G5	0.34	0.18	−2.31	−0.97 ^*∗*^	−0.41	−0.16	−0.50	0.25
G2 × G3	0.16 ^*∗∗*^	0.09 ^*∗∗*^	0.60 ^*∗∗*^	2.62 ^*∗∗*^	0.05	0.07 ^*∗∗*^	1.21 ^*∗*^	−0.38 ^*∗∗*^
G2 × G4	−0.26	−0.11	0.36	−0.16	0.13	1.16	−0.15	0.19
G2 × G5	0.02	0.06	−1.51	−1.49 ^*∗∗*^	−0.34	−1.06	−0.62	−0.13
G3 × G4	0.17 ^*∗∗*^	0.24 ^*∗∗*^	1.70 ^*∗∗*^	1.14 ^*∗∗*^	0.24	−0.98	0.59 ^*∗∗*^	−0.03 ^*∗∗*^
G3 × G5	−1.02 ^*∗∗*^	−0.43 ^*∗∗*^	−7.44 ^*∗∗*^	−4.44 ^*∗∗*^	−0.21	2.94	−1.80 ^*∗∗*^	−0.10 ^*∗∗*^
G4 × G5	0.23	0.00	−2.22	−1.37 ^*∗*^	−0.68 ^*∗*^	0.69	−1.13	0.03
SE (Sij)	0.18	0.10	1.09	0.65	0.12	0.71	0.29	0.19
SE (Sij-Sik)	0.27	0.15	1.67	0.99	0.18	1.10	0.44	0.29
SE (Sij-Skl)	0.24	0.13	1.45	0.86	0.15	0.95	0.38	0.25

*Reciprocal effect*								
G2 × G1	0.01	0.05	0.62	−0.08	0.07	1.35	0.01	0.10
G3 × G1	0.02 ^*∗∗*^	0.22 ^*∗∗*^	3.62 ^*∗∗*^	0.05 ^*∗∗*^	0.08	1.55 ^*∗∗*^	0.03 ^*∗*^	−0.32 ^*∗∗*^
G3 × G2	−0.05 ^*∗∗*^	0.02 ^*∗∗*^	−1.17	1.15 ^*∗∗*^	0.18	−0.55	0.41 ^*∗*^	−0.01 ^*∗∗*^
G4 × G1	0.15	−0.03	1.78	−0.15	−0.07	1.35	−0.32	−0.12
G4 × G2	0.15	0.07	0.30	0.35		0.58	−0.14	−0.04
G4 × G3	0.12 ^*∗∗*^	0.17	0.52 ^*∗∗*^	−0.37 ^*∗∗*^	0.12 ^*∗∗*^	0.68 ^*∗∗*^	−0.51	0.02 ^*∗∗*^
G5 × G1	0.17	0.10	2.92	1.20	0.08	0.50	0.75	0.05
G5 × G2	0.05	0.10	1.92	0.25 ^*∗∗*^	0.15	−8.27	−0.11	−0.43 ^*∗∗*^
G5 × G3	0.25 ^*∗∗*^	0.13 ^*∗∗*^	−1.47 ^*∗∗*^	−0.28 ^*∗∗*^	−0.22	7.42	−0.41 ^*∗∗*^	0.02 ^*∗∗*^
G5 × G4	0.02	−0.15	0.23	−0.07 ^*∗*^	−0.22 ^*∗*^	−0.27	−0.48	−0.13
SE Rij	0.22	0.12	1.32	0.78	0.14	0.87	0.35	0.23
SE (Rij-Rkl)	0.31	0.17	1.87	1.11	0.20	1.23	0.49	0.33

## Data Availability

The data used to support the findings of this study are available from the corresponding author upon request.
